# A dynamic pathway analysis approach reveals a limiting futile cycle in *N*-acetylglucosamine overproducing *Bacillus subtilis*

**DOI:** 10.1038/ncomms11933

**Published:** 2016-06-21

**Authors:** Yanfeng Liu, Hannes Link, Long Liu, Guocheng Du, Jian Chen, Uwe Sauer

**Affiliations:** 1 Key Laboratory of Industrial Biotechnology, Ministry of Education, School of Biotechnology, Jiangnan University, Lihu Road 1800, Wuxi 214122, China; 2 Key Laboratory of Carbohydrate Chemistry and Biotechnology, Ministry of Education, School of Biotechnology, Jiangnan University, Lihu Road 1800, Wuxi 214122, China; 3 Institute of Molecular Systems Biology, ETH Zurich, Zurich 8093, Switzerland; 4 Max Planck Institute for terrestrial Microbiology, Marburg 35043, Germany

## Abstract

Recent advances in genome engineering have further widened the gap between our ability to implement essentially any genetic change and understanding the impact of these changes on cellular function. We lack efficient methods to diagnose limiting steps in engineered pathways. Here, we develop a generally applicable approach to reveal limiting steps within a synthetic pathway. It is based on monitoring metabolite dynamics and simplified kinetic modelling to differentiate between putative causes of limiting product synthesis during the start-up phase of the pathway with near-maximal rates. We examine the synthetic *N*-acetylglucosamine (GlcNAc) pathway in *Bacillus subtilis* and find none of the acetyl-, amine- or glucose-moiety precursors to limit synthesis. Our dynamic metabolomics approach predicts an energy-dissipating futile cycle between GlcNAc6P and GlcNAc as the primary problem in the pathway. Deletion of the responsible glucokinase more than doubles GlcNAc productivity by restoring healthy growth of the overproducing strain.

## Abstract (web summary)

Rate-limiting steps in synthetic metabolic pathways are difficult to identify. Here, the authors monitor metabolite dynamics and apply kinetic modelling during the start-up phase of the *Bacillus subtilis* GlcNAc pathway to discover a futile cycle, allowing them to identify a more productive strain.

## Graphical abstract

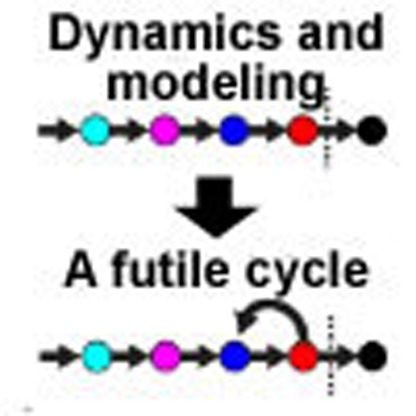

Metabolic engineering is a key technology for cellular property improvement in the green and sustainable production of biofuels, fine chemicals and pharmaceuticals^1^,^2^. The past 5 years have revolutionized our ability to engineer genomes, enabling high-precision strain construction at large-scale, for instance through multiplex automated genome engineering^3^, Cas9-mediated genome editing^4^ or modular pathway engineering^5^. Although these methods effectively removed all limitations for manipulating existing pathways and constructing synthetic ones to desired products, they further widened the already large gap between our ability to identify the engineering targets in the first place and precise diagnosis of arising bottlenecks and other complications. In other words: What is limit of production and how can we abolish the limitation?

The current repertoire to answer these questions ranges from computational methods that are often based on genome-scale models^6^,^7^,^8^ to omics methods that monitor cellular responses at the level of molecular component concentrations or metabolic fluxes^9^,^10^,^11^,^12^,^13^,^14^. A particular problem is diagnosis of rate-limiting steps in supply or within synthetic pathways from omics-type data. As most diagnosis tools are rather time-consuming, typical applications focus on steady-state analyses, such as transcriptome-based identification of an intermediate for pool size increase to improve riboflavin production^10^, proteome-based identification of two rate-limiting steps in isopentenol synthesis^11^ or metabolomics-based identification of an optimal intermediate feeding approach to improve tacrolimus production^12^. In particular, dynamic metabolomics has great diagnostic potential as was elegantly demonstrated by bottleneck identification within *in vitro* reconstituted glycolysis^15^ and a new method for near real-time metabolomics^16^. Surprisingly, applications of such dynamic investigations to biotechnological production systems are rare, presumably because design of meaningful experiments and data interpretation are not trivial. Consequently, diagnosis has so far largely been restricted to *ad hoc* identification of limitations in precursor supply that can easily be validated by precursors feeding without fundamentally optimizing structure and kinetic parameters of synthetic networks. Examples include identification of bottlenecks in biosynthesis pathways for lipids, recombinant antibodies and heterologous proteins^17^,^18^,^19^, and substrate degradation in xylose-fermenting *Saccharomyces cerevisiae*^20^.

Here we show that metabolite dynamics during the ‘start-up phase’ of a synthetic pathway with near maximal initial rates provide valuable information about limitations. We identify potential pathway bottlenecks from the comparison of metabolomics data with simulations of a simple kinetic model that can be generically applied to linear pathways. Specifically, we examined *Bacillus subtilis* metabolism during production of the amino sugar *N*-acetylglucosamine (GlcNAc) used for pharmaceutical and nutraceutical treatment of osteoarthritis and maintaining joint health^21^. The previously engineered strain with optimized GlcNAc synthesis and blocked GlcNAc catabolism^21^,^22^ (Fig. 1a) is productive in complex media but has poor performance and growth in industrial media with single carbon sources. The combination of dynamic metabolomics and coarse-grained modelling identified a major bottleneck and a potential metabolic engineering target, namely, the reactions between GlcNAc6P and GlcNAc, for improved cell growth and GlcNAc yield on glucose. Dynamic isotope tracing then diagnosed an energy-dissipating phosphorylation/dephosphorylation cycle as the molecular cause. Disrupting this futile cycle through deleting the encoding gene of the here newly identified responsible enzyme, glucokinase GlcK, restored intracellular GlcNAc6P concentrations and more than doubled GlcNAc productivity and GlcNAc yield on glucose.

## Results

### GlcNAc overproducing *B. subtilis* on minimal glucose medium

**Figure 1:**
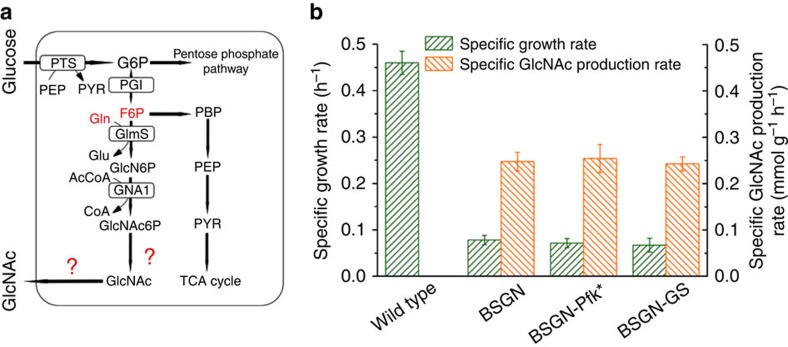
GlcNAc synthesis pathway and physiological comparison of wild-type *Bacillus subtilis* 168 and engineered GlcNAc-producing *B. subtilis*. (**a**) Metabolic pathway of GlcNAc synthesis. (**b**) Comparison of specific cell growth rate and specific GlcNAc production rate of *B. subtilis* 168, the GlcNAc-overproducing *B. subtilis* BSGN6-P_*xylA*_-*glmS*-P_*43*_-*GNA1* (BSGN) strain, BSGN with mutation in 6-phosphofructokinase (BSGN-Pfk*), BSGN with glutamate synthase overexpression (BSGN-GS). AcCoA, acetyl-coenzyme A; DHAP, dihydroxyacetone phosphate; F6P, fructose-6-phosphate; FBP, fructose-1,6,-bis-phosphate; G6P, glucose-6-phosphate; GlcN6P, glucosamine-6-phosphate; GlcNAc6P, *N*-acetylglucosamine-6-phosphate; GlmS, glucosamine synthase; Gln, glutamine; Glu, glutamate; GNA1, GlcN6P *N*-acetyltransferase; PEP, phosphoenolpyruvic acid; PGI, glucose-6-phosphate isomerase; PTS, phosphotransferase system; PYR, pyruvate; OD_600_, optical density at 600 nm. Triplicate experiments were done for physiological measurements, and error bars represent standard deviation.

An engineered GlcNAc production strain *B. subtilis* BSGN6-P_*xylA*_-*glmS*-P_*43*_-*GNA1*, abbreviated BSGN hereafter, was recently obtained based on modular pathway optimization to fine-tune a synthetic GlcNAc pathway in our previous research^21^. In brief, BSGN was constructed by overexpression of glucosamine (GlcN)-6-phosphate synthase (GlmS) under the control of an inducible promoter P_*xylA*_ and GlcN-6-phosphate *N*-acetyltransferase (Gna1) under the control of a constitutive promoter P_*43*_ in the background of absent GlcNAc catabolism via knockout of *nagP*, *gamP*, *nagA*, *nagB* and *gamA*. In glucose minimal medium, the growth rate of BSGN was only a fifth of the parent strain (Fig. 1 and Supplementary Table 1). Because GlcNAc production is coupled to cell growth, GlcNAc productivity dropped dramatically by 82.7 % to 32.6 mg g^−1^ dry cell weight per hour in glucose minimal medium with a yield of 65.0 mg GlcNAc per gram glucose. Here we address the problem of cell growth and product formation in the industrially relevant condition of glucose minimal medium. Given that GlcNAc overproduction requires three central metabolic precursors (fructose-6-P, acetyl-CoA (AcCoA) and the nitrogen donor glutamine), competition between growth-related metabolism and the synthetic pathway is expected. Hence, we first examined the effect of precursor supply on native metabolism and the synthetic pathway.

To probe whether precursor supply constrained either GlcNAc formation or native metabolism, we genetically modulated fluxes through the supply pathways. First, we diverted fructose-6-P availability from native metabolism to GlcNAc by replacing the native phosphofructokinase (Pfk) with a reduced activity of Arg252Ala-mutated Pfk^23^. Surprisingly, the mutation had no effect on growth and specific production rate of BSGN-Pfk* were similar to BSGN (Fig. 1b). Therefore, fructose-6-P supply neither limits GlcNAc production nor cell growth. Likewise, increased supply of glutamine for GlcNAc synthesis and central nitrogen metabolism in strain BSGN-GS by overexpression of glutamine synthase (GS) did not improve growth or GlcNAc production rate (Fig. 1b and Supplementary Table 2), but increased the specific glucose uptake rate about 1.5-fold relative to BSGN and BSGN-Pfk*. Also addition of glutamine to the cultivation medium had no effect on specific growth rate and GlcNAc-specific production rate (Supplementary Fig. 1). Thus, we conclude that precursor supply would be sufficient for a much higher GlcNAc production and cell growth than observed in BSGN.

### Metabolomics reveals abundant precursor and bottleneck

**Figure 2:**
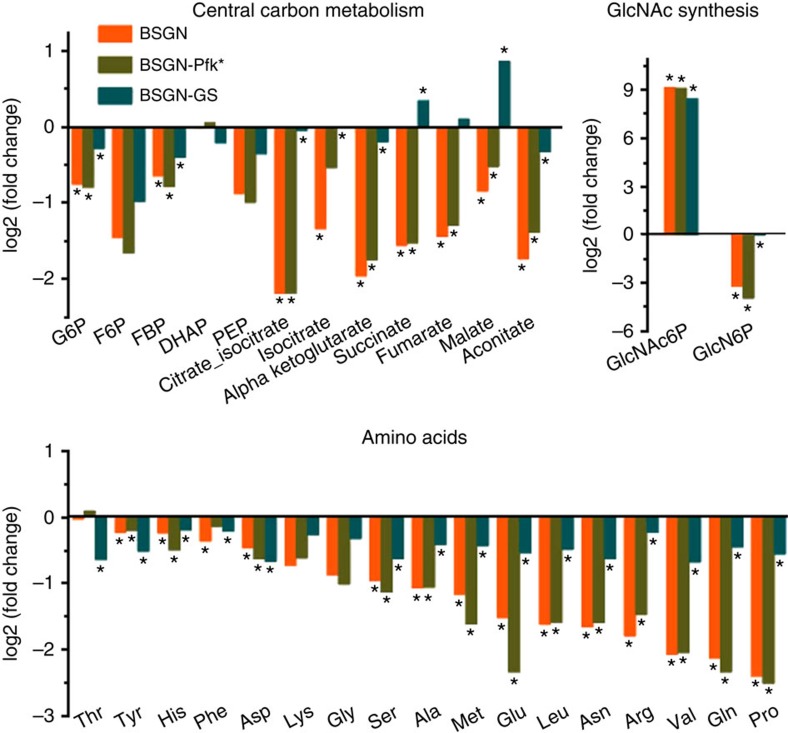
Metabolite pool size comparison of engineered *B. subtilis* to *B. subtilis* 168 wild-type strain. Pool sizes of metabolite in glycolysis, the TCA cycle, amino acids and amino sugars were compared. Triplicate experiments were done for metabolite pool size measurements. Asterisk (*) denotes *P*<0.05 (two-tailed test). The standard deviations values of all the fold changes of triplicate experiments were less than 20% of the value of fold changes.

To identify the primary limitation in GlcNAc biosynthesis, we determined intracellular metabolite concentrations in wild-type *B. subtilis*, the GlcNAc production strain BSGN and the two producing strains BSGN-Pfk* and BSGN-GS with modulated precursor supply during mid-exponential growth (at an optical density at 600 nm of 0.5) in minimal glucose medium. Almost all metabolite concentrations were lower in BSGN compared with wild-type, and, in particular, the GlcNAc pathway precursors fructose-6-P, citrate (directly connected to AcCoA) and the nitrogen donor glutamine were markedly decreased (Fig. 2, Supplementary Fig. 2 and Supplementary Tables 3 and 4). How did modifications of precursor supply change this profile? Although the Pfk mutation in BSGN-Pfk* had no further effect on metabolite concentrations, GS overexpression in BSGN-GS restored nearly wild-type levels for most metabolites, suggesting that GlcNAc production impaired cellular nitrogen metabolism, presumably through low levels of glutamine. Nevertheless, BSGN-GS grew as slowly as the ancestral BSGN despite restored metabolite levels (Fig. 1b). Thus, the decreased metabolite concentrations were neither consequence nor major cause of slow growth, strongly suggesting that other mechanisms impair growth and GlcNAc synthesis.

The essentially only and very striking difference between all three production strains and wild type was a more than 300-fold increased GlcNAc-6-P concentration from 0.06 mM in the wild type to 20–34 mM in the GlcNAc-producing strains (Fig. 2). These abnormal concentrations suggest a metabolic bottleneck in the vicinity of GlcNAc6P. As the concentration of phosphosugars is normally tightly controlled, such extraordinarily high GlcNAc6P concentrations may cause phosphosugar stress, affecting for instance nucleic acid metabolism and triggering degradation of *ptsG* messenger RNA regulated by the small RNA SgrS at the posttranscriptional level, which, in turn, inhibits glucose uptake in *E. coli*^24^,^25^,^26^. Based on steady-state data alone, however, one cannot differentiate between different molecular causes such as, for example, low activity of the dephosphorylating enzyme or transport limitations that jam the pathway.

### Different bottlenecks lead to distinct metabolite dynamics

**Figure 3:**
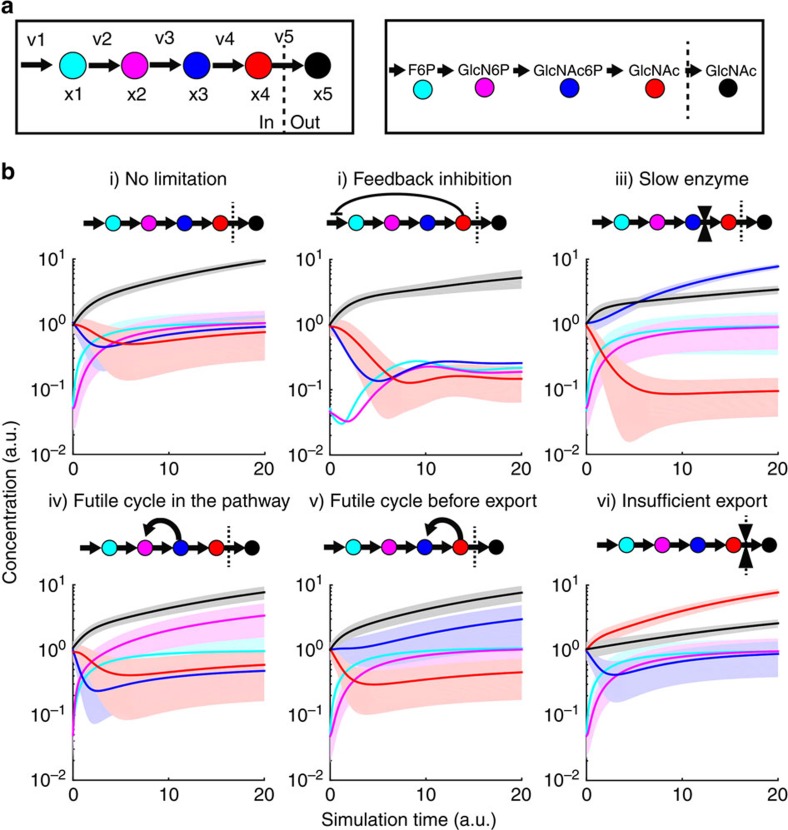
Schematic representation of the dynamic metabolome-based pathway bottleneck identification. (**a**) A linear production pathway with five reactions (*v*1–*v*5), four intracellular metabolites (*x*1-*x*4) and one extracellular product *x*5. (**b**) Simulation results of the linear pathway assuming different scenarios: (i) no limitation in the pathway; (ii) feedback inhibition in the pathway; (iii) a rate-limiting step in the pathway; (iv) a futile cycle in the pathway; (v) a futile cycle at the end of the pathway or (vi) insufficient intracellular product export in the pathway. One hundred simulation results using 100 random parameter sets for initial concentrations and *K*_m_ values (lines indicate mean and shaded areas the standard deviation). Colours are as shown in above in **a**. The model is described in detail in the Methods section.

Given that metabolite levels respond very fast, we reasoned that they would be a sensitive indicator of arising bottlenecks during a transition from low to high activity of the synthetic pathway and we sought to test putative dynamics using a metabolic model^16^,^27^ (Fig. 3a). For this purpose, we developed a linear pathway model, which consists of three intermediate metabolites, the intracellular and the extracellular products. To simulate a transition from low to high pathway activity, initial concentrations of the first two intermediates are randomly sampled from a sub-saturating concentration range (between 0 and 10% of average *K*_m_ values). Initial concentrations of the other three metabolites are randomly sampled from a near-saturating concentration range (50 and 150 % of average *K*_m_ values). All reactions are described by Michaelis–Menten kinetics and *K*_m_ values are randomly sampled between 0 and 2, resulting in an average *K*_m_ of one. Concentrations of metabolites and *K*_m_ parameters are arbitrary units but their range account for low and high saturating conditions of enzymes. In a base model, all maximal reaction rates were fixed to 1, except influx which was 0.5 in order to assuring that no reaction in the pathway is limiting. Simulations of the base model (i) show a continuous increase of product and asymptotic equilibration of intermediates at the average *K*_m_ of one (Fig. 3b). Next, we simulated possible scenarios of frequently encountered pathway limitations, including end-product inhibition, as well as futile cycles and limiting enzyme capacities at several nodes of the pathway (Fig. 3b). In case of feedback inhibition (ii), concentrations of intermediates equilibrate at average inhibition constants and overproduction are reduced. (iii) A typical bottleneck reaction with low turnover has a continuously accumulating intermediate upstream of the bottleneck, whereas the downstream intermediate decreases strongly below the average *K*_m_ value. In case of the futile cycles shown in scenario (iv) and (v), the dynamic response is similar to a limiting reaction in scenario (iii). However, intermediates up- and downstream of the futile cycle equilibrate faster than in case of a limiting reaction. Quantitatively, the response of intermediates depends on the degree of futile cycle and the bottleneck, with much stronger responses in the latter case (Supplementary Fig. 3). Finally, if product export does not match the synthesis rate the intracellular product accumulates (vi). Thus, although metabolite dynamics are difficult to predict quantitatively, their qualitative features bear sufficient information to distinguish between different putative bottlenecks.

**Figure 4:**
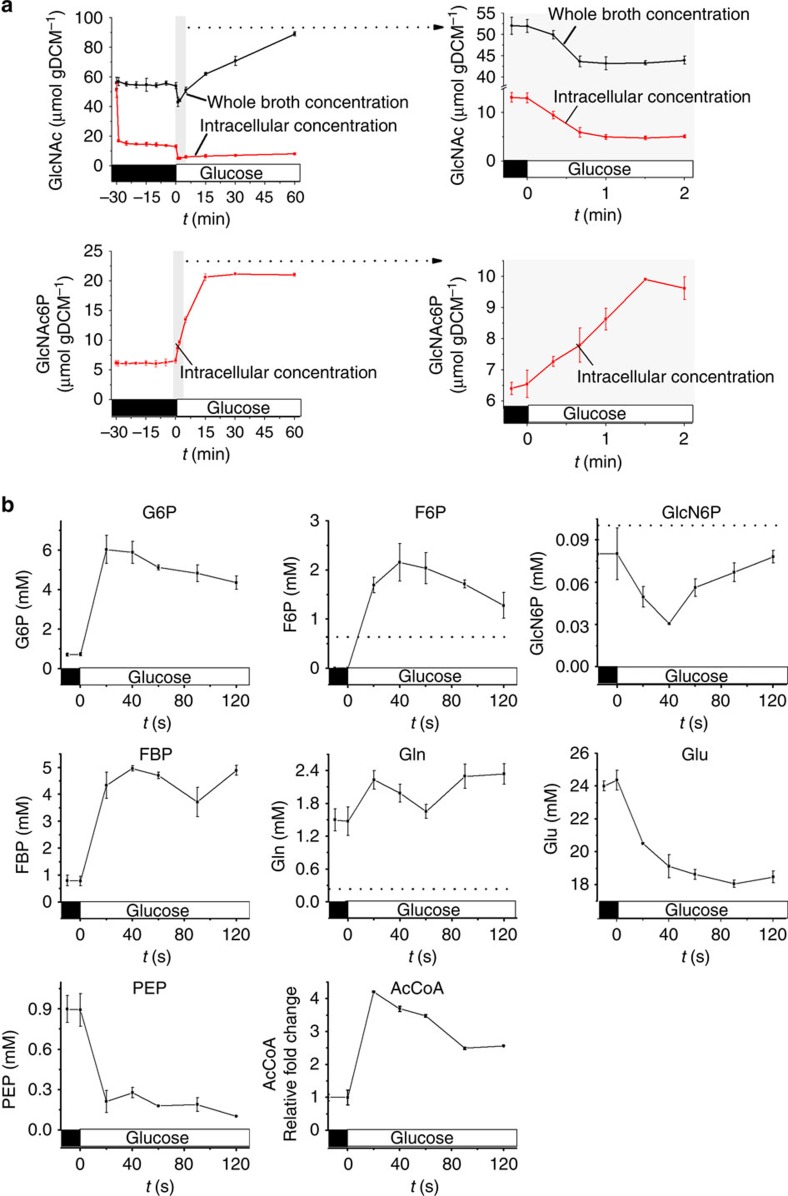
Metabolite dynamics in the GlcNAc synthetic pathway. (**a**) Dynamic experiment setup and dynamic change of GlcNAc and GlcNAc6P. Cells were harvested by centrifugation and resuspended in M9 medium without any carbon source. Next, cells were incubated in the no carbon source condition for 30 min until depletion of substrate of synthetic pathway and no further change of GlcNAc was observed. Glucose was then added to initiate GlcNAc synthesis (*t*=0). Intracellular metabolite concentrations and metabolite concentrations in whole-cell broth were analysed. DCW, dry cell weight. (**b**) Dynamics of intracellular metabolite in glycolysis and GlcNAc synthetic pathway by adding glucose was added as substrate at *t*=0. The known *K*_m_ values of pathway enzymes for F6P, GlcN6P and Gln were shown by dashed lines. Triplicate experiments were done for metabolite dynamics analyses, and error bars represent standard deviation.

To experimentally realize a transition from low to high activity of the GlcNAc pathway, exponentially growing cells were harvested and resuspended in medium without carbon source. Low precursor availability was ensured by 30 min incubation without carbon source that effectively stopped GlcNAc production and lead to constant intra- and extracellular metabolite concentrations without significant changes in pathway enzyme expression (Fig. 4a and Supplementary Figs 4 and 5). Addition of glucose induced GlcNAc synthesis at a rate of 34.8 μmol g^−1^ dry cell weight per hour that remained constant for 1 h (Fig. 4a). Monitoring metabolite dynamics during the first 2 min after glucose addition revealed the known response of glycolytic intermediates to glucose; that is, rapid increase of phosphorylated sugars and decrease of the phosphotransferase system substrate phosphoenolpyruvic acid (Fig. 4b). Concentrations of the three GlcNAc precursors fructose-6-P and glutamine increased rapidly above the *K*_m_ values of their cognate enzymes^25^,^28^ (Fig. 4b and Supplementary Table 5), confirming our hypothesis of absent limitations in the precursor supply into the pathway. Within the simulated responses of possible scenarios, the scenario with a futile cycle or a limiting reaction at the end of the pathway would be consistent with the experimentally detected dynamics (Figs 3b and 4b). As GlcNAc6P concentrations equilibrate already after 2 min and intracellular GlcNAc remained high (5 mM), we hypothesized that a futile cycle is more likely than a limiting dephosphorylation (Fig. 3 and Supplementary Fig. 3). Next, we sought to investigate if indeed the product GlcNAc is re-phosphorylated by an unknown enzyme, resulting in an ATP dissipating futile cycle.

### Validation and preventing the ATP dissipating futile cycle

**Figure 5:**
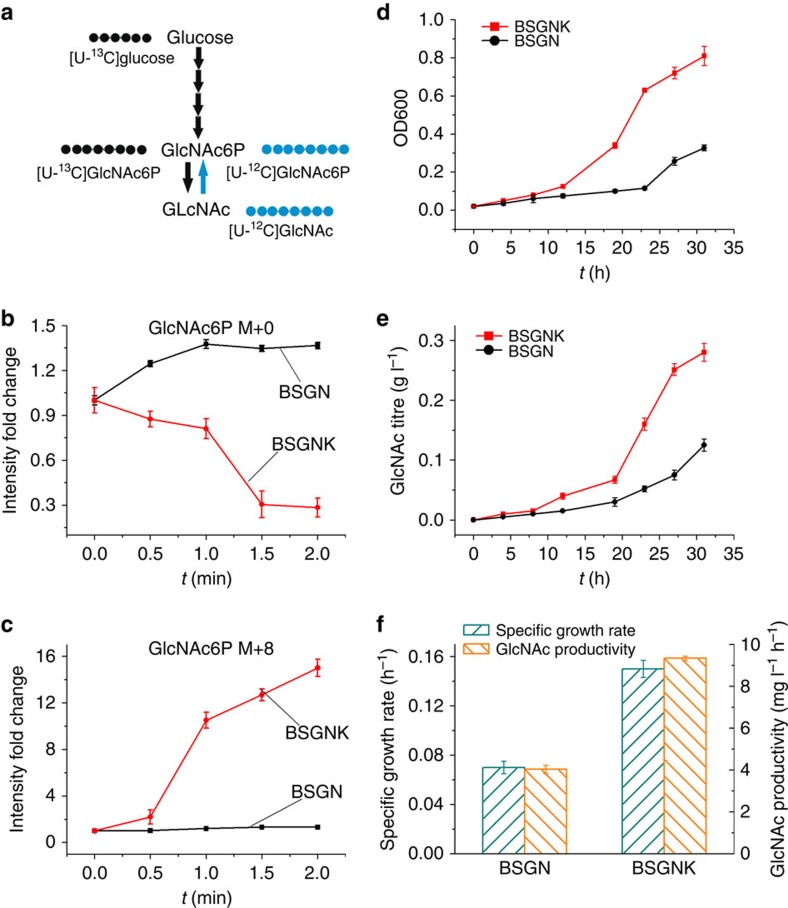
Dynamic labelling of GlcNAc6P using [U-^13^C]glucose as a substrate. (**a**) Labelling patterns of GlcNAc6P with substrate from [U-^13^C]glucose and GlcNAc, respectively. Black circles indicate ^13^C pattern carbon atoms; blue circles indicate ^12^C pattern carbon atoms. (**b**) Comparison of intensity fold change of GlcNAc6P mass isotopomer GlcNAc6P M+0 and GlcNAc6P M+8 after [U-^13^C]glucose addition in BSGN and BSGN with glucokinase encoding gene (*glcK*) deletion (BSGNK). (**c**) Comparison of intensity fold change of GlcNAc6P mass isotopomer GlcNAc6P GlcNAc6P M+8 after [U-^13^C]glucose addition in BSGN and BSGNK. (**d**) Comparison of cell growth in BSGN and BSGNK in a shake flask production system. (**e**) Comparison of GlcNAc production in BSGN and BSGNK in a shake flask production system. (**f**) Comparison of specific cell growth rate and GlcNAc productivity of BSGN and BSGNK strain in a shake flask production system. Triplicate experiments were done for dynamic labelling of GlcNAc6P, and error bars represent standard deviation.

To experimentally validate the above-hypothesized futile cycle, we used isotopic tracer experiments. As intracellular GlcNAc decreased upon pathway start-up (Fig. 4a), futile re-phosphorylation to GlcNAc6P should occur to a large extent from unlabelled GlcNAc molecules during the initial phase. To verify that the initial source of accumulating GlcNAc6P was indeed GlcNAc rather than glucose via the normal synthesis route, we started the experiment with uniformly labelled [U-^13^C]glucose (Fig. 5a). Within the first 60 s of [U-^13^C]glucose addition, the relative concentration of unlabelled [U-^12^C]GlcNAc6P (M+0) increased 36% (Fig. 5b), whereas no fully labelled [U-^13^C]GlcNAc6P was yet formed (Fig. 5c), demonstrating that re-phosphorylation dominates during this period. Thus, an energy-dissipating phosphorylation/dephosphorylation futile cycle operates between GlcNAc6P and GlcNAc, effectively blocking GlcNAc synthesis. The resulting accumulation of GlcNAc6P may further trigger phosphosugar stress and impair cell growth and GlcNAc production. Alleviating GlcNAc6P accumulation and improving the GlcNAc production would require disruption of the futile cycle between GlcNAc and GlcNAc6P. However, a GlcNAc kinase has so far not been annotated in *B. subtilis*. To identify candidate genes encoding such a GlcNAc kinase, we performed homology analysis using the amino-acid sequence of *E. coli* GlcNAc kinase NagK^29^,^30^. Among the 106 kinases in *B. subtilis*, the highest homology was found for the annotated glucokinase GlcK (26% sequence identity), and we deleted *glcK* in the BSGN strain (yielding strain BSGNK). Repeating the dynamic labelling experiment with BSGNK abolished formation of GlcNAc6P M+0 and labelled GlcNAc6P M+8 increased strongly, because of *de novo* synthesis from glucose (Fig. 5c). Thus, we conclude that deletion of *glcK* resulted in drastic reduction of the futile cycle.

Beyond the short-term dynamics, we next investigated GlcNAc synthesis in BSGNK under more production relevant conditions in steady-state cultures grown in minimal medium with glucose. Indeed, breaking the futile cycle through the *glcK* deletion circumvented the detrimental increase of intracellular GlcNAc6P (0.06 mM in BSGNK versus 33.71 mM in BSGN, Supplementary Fig. 6). The decrease of intracellular GlcNAc6P relieved potential phosphosugar stress for the cell and restored a more healthy growth physiology with a more than doubled specific cell growth rate and more than doubled GlcNAc productivity (9.20 mg l^−1^ h^−1^; Fig. 5d–f). Therefore, the *glcK* deletion experiments confirmed the deleterious role of the futile cycle between GlcNAc6P and GlcNAc for cell growth and GlcNAc production in engineered *B. subtilis*. Moreover, the energy charge increased from 0.68±0.03 in BSGN to 0.81±0.04 in BSGNK (+/− values represent standard deviation from triplicate experiments), further confirming that eliminating the ATP-dissipating futile cycle improved energy metabolism (Supplementary Fig. 7). Moreover, the GlcNAc yield on glucose (147.5 mg g^−1^ glucose) and dry cell weight on glucose (138.3 mg g^−1^ glucose) in BSGNK were 2.3-fold higher than in BSGN. As the specific GlcNAc production rate was similar in BSGN (32.6 mg g^−1^ DCW h^−1^) and BSGNK (33.2 mg g^−1^ DCW h^−1^), improved performance of BSGNK resulted from restored healthy growth with a high growth rate, which is apparently coupled to GlcNAc production (Supplementary Table 1).

## Discussion

Identification of bottlenecks within engineered pathway still hampers rational metabolic engineering. To differentiate between limitations within a synthetic pathway or in precursors supply to it, we first used comparative steady-state metabolomics profiling of genetically perturbed supply pathway strains. To hypothesize bottlenecks within a synthetic pathway, we outlined an approach based on dynamic metabolomics experiments and simplified kinetic modelling to differentiate between different putative causes in limiting GlcNAc synthesis during the start-up phase of the pathway with near maximal rates. The potential of this generically applicable approach was demonstrated by identifying an unexpected futile phosphorylation–dephosphorylation cycle in the GlcNAc production with *B. subtilis*, genetic intervention of which greatly improved GlcNAc productivity and yield on glucose. Relieving the energy drain induced by this synthetic pathway restored the cellular energy homeostasis and healthy growth and thus in consequence volumetric GlcNAc productivity.

## Methods

### Strains and plasmids and cultivation

Strains and plasmids used in this study are listed in Supplementary Table 6. The previously constructed GlcNAc production strain BSGN^21^ is characterized by (i) a block of GlcNAc catabolism through marker-free deletion of all relevant encoding genes and (ii) overexpression of the GlcNAc synthesis enzymes GlmS and GNA1. BSGN-Pfk* was constructed by introducing site-directed mutation in native *pfk* Arg252Ala via a markerless genome editing system^31^ for *B. subtilis* with the primers listed in Supplementary Table 7. In brief, front and back homology fragments with the mutation (Arg252Ala, CGC to GCC) in direct repeated sequences were amplified with primers AL-F/AL-R and AR-F/AR-R, respectively. The *mazF* cassette was amplified with primers AZ-F and AZ-R from the *B. subtilis* 168 genome. Next, fusion PCR was performed to fuse the front homology fragment, *mazF* cassette, and back homology fragment. The resulting DNA fragment was transformed into BSGN0 via screening on a Luria–Bertani (LB) plate with spectinomycin. Transformants with mutation site integration were then plated on LB plates with 2% xylose to screen strains with the desired mutation and *mazF* cassette eviction via single-crossover between directed repeated sequences. To construct BSGN-GS, we constructed DNA multimer plasmids pP43-GNA1-GS via DNA multimer-based vector construction manipulation^32^. In brief, encoding sequences of GNA1 and GS were amplified with primers GNA1-F/GNA1-R and GS-F/GS-R from plasmid pP43-GNA1 and the *B. subtilis* 168 genome. For encoding sequences of GNA1, 3′ overlapped fragment of the vector sequence was introduced. For encoding sequences of GS, 5′ overlapped fragment of the vector sequence was introduced. The vector sequence was amplified with PCR using primers V-F and V-R. The resulting encoding sequences of GNA1 and GS were fused via fusion PCR. Prolonged-overlap extension PCR was then performed with the GNA1-GS and vector fragments to generate DNA multimer plasmids, which were transformed into BSGN0 via multimer plasmid cleavage of the host strain forming a circular plasmid and yielding BSGN-GS. BSGNK was constructed by knockout of glucose kinase encoding gene sequence *glcK*. In brief, primers GlcK-F/GlcK-R were used to amplify *glcK* disrupt cassette from *B. subtilis* 168 *ΔglcK* (Professor Jörg Stülke, Georg-August-Universität Göttingen). The amplified *glcK* disrupt cassette was transformed into BSGN, yielding BSGNK.

Cultivation for genetic experiments was done in LB broth or agar plates (10 g l^−1^ tryptone, 5 g l^−1^ yeast extract and 10 g l^−1^ NaCl) at 37 °C. Cultivation for physiological experiments was done in M9 mineral salts medium with 2 g l^−1^ glucose (1 g l^−1^ NH_4_Cl, 0.5 g l^−1^ NaCl, 8.5 g l^−1^ Na_2_HPO_4_·H_2_O, KH_2_PO_4_, 1 ml of 1 M MgSO_4_ per litre, 1 ml of 0.1 M CaCl_2_ per litre, 1 ml of 0.05 M FeCl_3_ and 10 ml trace element solution containing 60 mg l^−1^ CoCl_2_·6H_2_O, 43 mg l^−1^ CuCl_2_·2H_2_O, 100 mg l^−1^ MnCl_2_·4H_2_O, 60 mg l^−1^ Na_2_MoO_4_·2H_2_O and 170 mg l^−1^ ZnCl_2_). Appropriate antibiotics were added to the medium at the following final concentrations: kanamycin (20 μg ml^−1^) and spectinomycin (100 μg ml^−1^). To evaluate effects of Gln feeding on cell growth and GlcNAc production, Gln was added to final concentration 0.5 mmol l^−1^ in M9 mineral salts medium.

### Steady-state targeted metabolomics

*B. subtilis* strains were cultured in M9 medium in a shake flask at 37 °C and 300 r.p.m. Cells were harvested in the mid-exponential phase when the optical density at 600 nm reached 0.5. Fast-filtration was used to collect cells on a filter, and the cells were further quenched and extracted in acetonitrile/methanol/H_2_O (40:40:20) solution with ^13^C internal standard addition. Next, the extract solution was dried and resuspended in H_2_O for ultrahigh-performance liquid chromatography-tandem mass spectrometry (UHPLC-MS/MS) detection using the following conditions^33^: Waters Acquity UHPLC (Waters Corporation) equipped with Waters Acquity T3 C18 column (150 × 2.1 × 1.8 μm^3^, Waters Corporation) was tandemed with Thermo TSQ Quantum Ultra triple quadrupole instrument (Thermo Fisher Scientific) with a heated electrospray ionization source (Thermo Fisher Scientific). Temperature of UHPLC column was controlled at 40 °C. Mobile phase A (10 mM tributylamine, 15 mM acetic acid, 5% (v/v) methanol) and B (2-propanol) were used for gradient elution for metabolite separation. The MS was operated in negative mode, and following parameters of MS were used: Ion spray voltage 2,500 V, ion sweep gas pressure 5 arbitrary units, auxiliary gas pressure 50 arbitrary units, curtain gas pressure 80 arbitrary units. Tube lens voltage, collision energy and fragment ions were optimized individually for all compounds^33^. Metabolite fold changes between recombinant strains and wild-type *B. subtilis* 168 were calculated based on metabolite concentrations.

### Metabolite dynamics analysis and dynamic labelling

Cells were cultivated in LB medium and harvested via centrifugation (4,500*g*, 5 min) when optical density at 600 nm reached 1.0. Next, the cells were resuspended in M9 medium without glucose and incubated at 37 °C with magnetic stirring at 400 r.p.m. At *t*=0, glucose was added to a final concentration of 2 g l^−1^. Then, samples of whole-cell broth for total metabolite concentration measurements, including both intracellular and extracellular concentrations, were taken after glucose addition and immediately quenched and extracted in acetonitrile/methanol/H_2_O (40:40:20) solution. Cells for intracellular metabolite concentration measurements were collected on a 0.45-μm pore size nitrocellulose filter membranes (Millipore). ^13^C internal standard was added before the samples were dried. After sample resuspension in H_2_O, UHPLC-MS/MS was performed to detect metabolites^33^.

For the dynamic labelling experiment, 100% [U-^13^C]glucose was used as the substrate. Data of mass isotopomers GlcNAc6P M+0 and M+8 were acquired via Xcalibur software version 2.07 SP1 (Thermo Fisher Scientific). Peak integrations were performed with Xcalibur software version 2.07 SP1 (Thermo Fisher Scientific) and a Matlab R2014b (The Mathworks Inc.)-based in-house integration software for intensity calculation.

### Dynamic simulation

MATLAB R2014b was used for all calculations and the dynamic simulation. Base Model (Model 1—no limitation): The linear pathway is shown in Fig. 3a in the main text. It consists of four intracellular metabolites *x*_(1)_–*x*_(4)_ and the extracellular product *x*_(5)_. Reaction kinetics of five reactions are:

Influx of precursor:


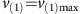


Michaelis–Menten kinetics are used to describe reactions kinetics of reaction *v*_(2)_, *v*_(3)_, *v*_(4)_ and *v*_(5)_:


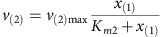



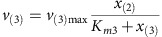



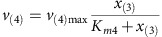



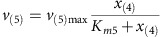


Mass balances for all metabolites result in the differential equations:


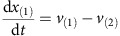



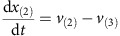



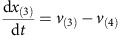



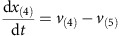



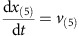


Parameters: The maximum reaction rates *v*_(2)max_, *v*_(3)max_, *v*_(4)max_ and *v*_(5)max_ are 1 and *v*_(1)max_ is 0.5. The binding constants *K*_*m*2_, *K*_*m*3_, *K*_*m*4_, *K*_*m*5_ were randomly sampled between 0 and 2. The initial concentrations of *x*_(1)_ and *x*_(2)_ were sampled between 0 and 0.1, initial concentrations of *x*_(3)_, *x*_(4)_ and *x*_(5)_ were sampled between 0.5 and 1.5.

The models for the different scenarios (ii)–(vi) are:

Model 2: To describe the scenario with feedback inhibition (Fig. 3b), intracellular product *x*_(4)_ inhibits the first reaction *v*_(1)_, according to Hill kinetics. The Hill coefficient *n* was sampled between 0 and 4, and *K*_*i*_ between 0 and 1.


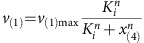


Model 3: To describe the scenario with a limiting enzyme abundance *v*_(4)max_ was 0.1, and in Supplementary Fig. 3 as indicated.

Model 4 and model 5: To describe the scenario with a futile cycle, an additional reaction was added *v*_(6)_, described again by Michaelis–Menten kinetics. The binding constants *K*_*m*6_ was randomly sampled between 0 and 2.


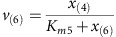


*v*_(6)max_ was 1 in Fig. 3, and in Supplementary Fig. 3 as indicated.

Model 6: To describe the scenario with insufficient intracellular product exportation *v*_(5)max_ was 0.1.

### Transcriptional level comparison of GlcNAc pathway enzymes

The following cells are harvested, frozen in liquid nitrogen and stored in −80 °C freezer for RNA extraction and quantitative real-time PCR analysis: (i) exponentially growing BSGN cell in M9 minimum medium, (ii) exponentially growing BSGN cell in LB medium, (iii) exponentially growing BSGN cell in LB medium with resuspension in M9 minimum medium without glucose for 15 min substrate depletion and (iv) exponentially growing BSGN cell in LB medium with resuspension in M9 minimum medium without glucose for 30 min substrate depletion. Total RNA of above cells was extracted via Qiagen RNeasy Mini Kit (QIAGEN). cDNA was obtain for RNA using PrimeScrip RT reagent Kit (Takara). Primers used for qRT–PCR were listed in Supplementary Table 6. LightCycler 480 II real-time PCR instrument (Roche Applied Science) was used to quantify cDNA with SYBR Premix Ex Taq (Takara). Gene expression levels of exponentially growing cell without incubation were defined as 100%. Relative gene expression changes were calculated based on normalized data to 16s rRNA. Triplicate experiments were done for relative gene expression assay.

### Data availability

The Matlab code used in this study and data that support the findings of this study are available from the corresponding author upon request.

## Additional information

**How to cite this article**: Liu, Y. *et al*. A dynamic pathway analysis approach reveals a limiting futile cycle in *N*-acetylglucosamine overproducing *Bacillus subtilis*. *Nat. Commun.* 7:11933 doi: 10.1038/ncomms11933 (2016).

## Supplementary Material

Supplementary Information Supplementary Figures 1-7 and Supplementary Tables 1-7
