# Supplementary material for: A dynamic pathway analysis approach reveals a limiting futile cycle in N-acetylglucosamine overproducing Bacillus subtilis
Source: Nat Commun. 2016 Jun 21;7:11933. doi: 10.1038/ncomms11933 (PMC5512609; doi:10.1038/ncomms11933)
Supplement: Supplementary Information — Supplementary Figures 1-7 and Supplementary Tables 1-7 [file ncomms11933-s1.pdf]

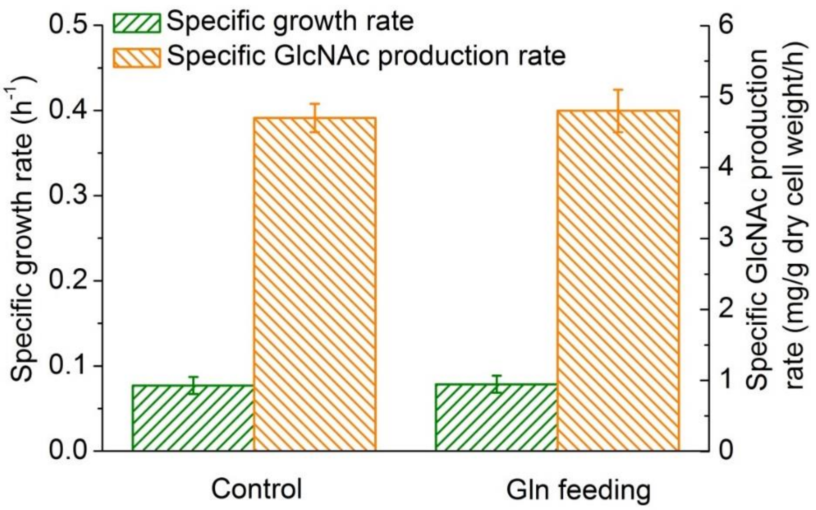

Supplementary Figure 1. Effects of glutamine feeding on cell growth and GlcNAc production for BSGN.

Control: M9 minimum medium without glutamine was used for shake flask fermentation for BSGN; Gln feeding: M9 minimum medium with 0.5 mM glutamine was used for shake flask fermentation for BSGN. Error bars represent standard deviation of triplicate experiments.

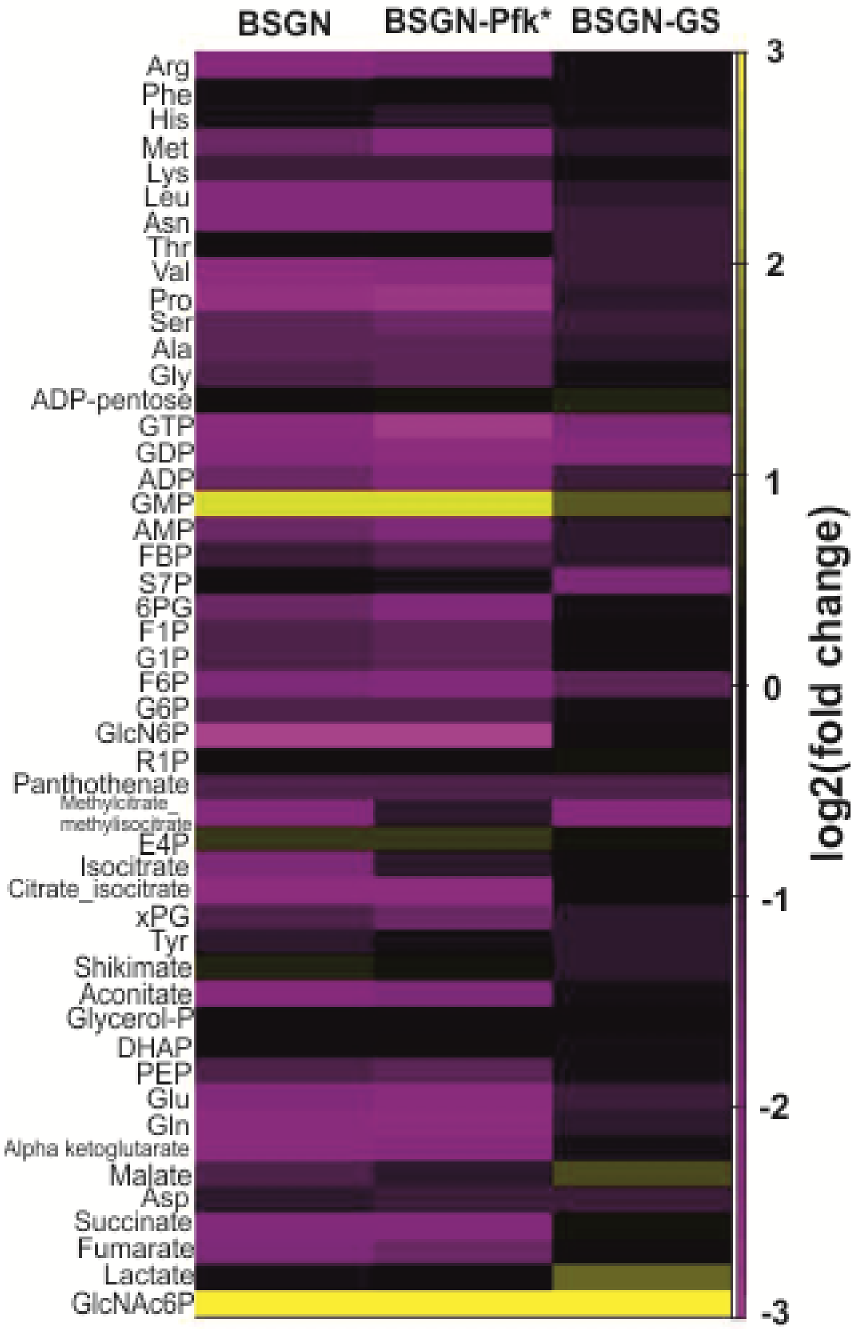

Supplementary Figure 2. Overview of fold changes of metabolites in central carbon and nitrogen metabolism in BSGN, BSGN-Pfk*, and BSGN-GS. All the standard deviations of metabolite concentrations of triplicate experiment are less than 20 %.

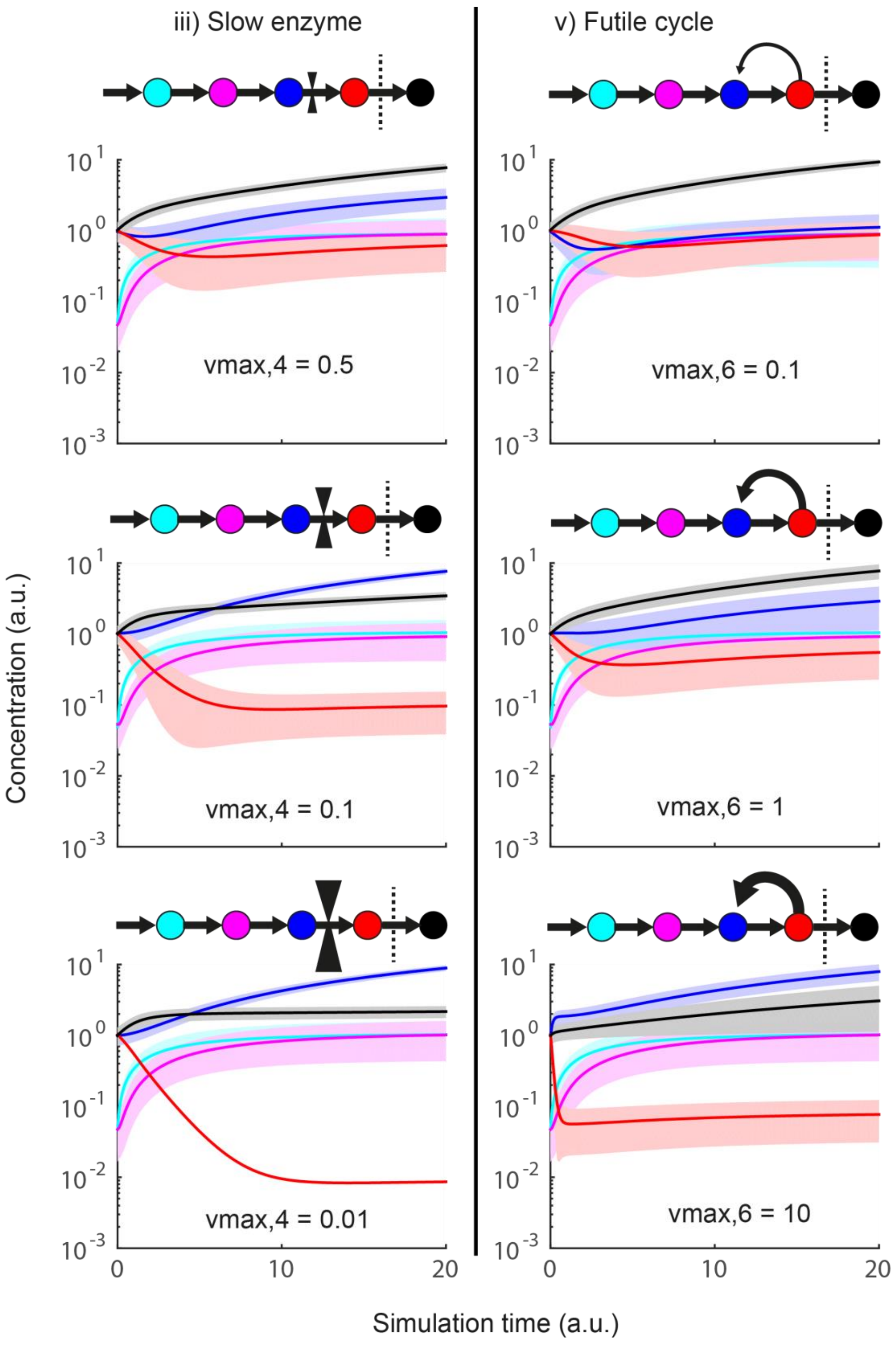

Supplementary Figure 3. Comparison of simulation results of slow enzyme and futile cycle in synthetic pathway with various v_{max} value of limiting reaction and "futile" reaction.

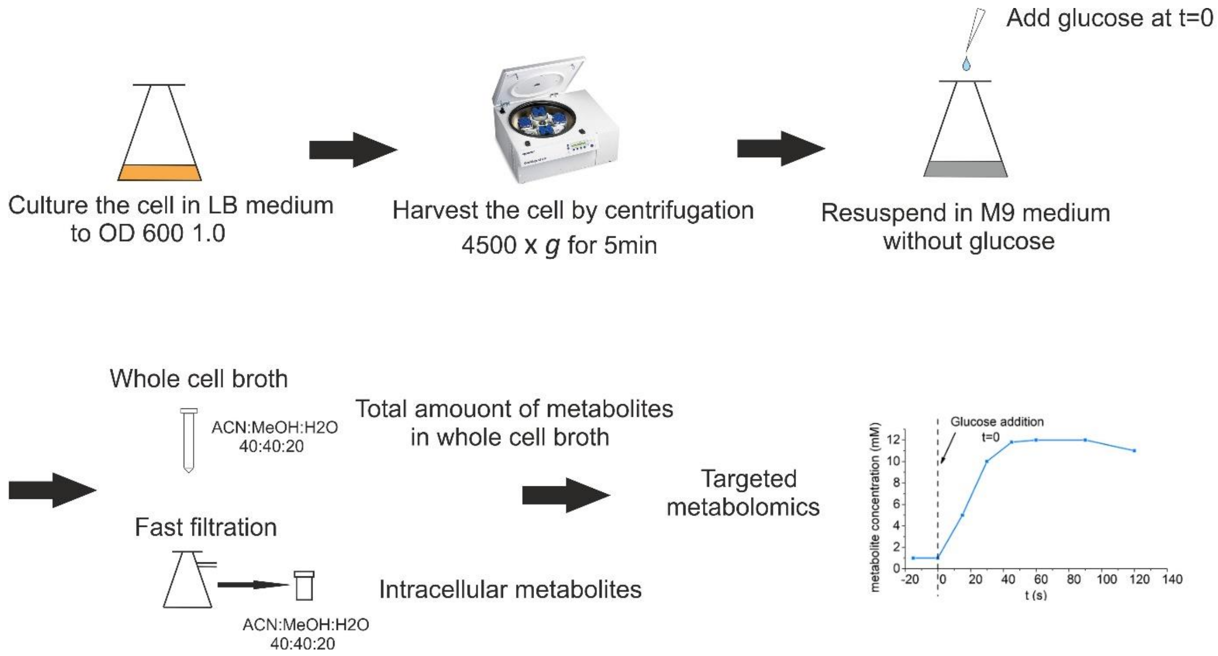

Supplementary Figure 4. Experimental setup for dynamic metabolite change analysis during start-up of GlcNAc synthesis. Cells were harvested by centrifugation and resuspended in M9 medium without any carbon source. Next, cells were kept in the no carbon source condition for 30 minutes until no further change of GlcNAc was observed. Glucose was then added to initiate GlcNAc synthesis. Metabolite concentrations in whole cell broth and intracellular concentrations were analyzed. ACN, acetonitrile; LB, Luria-Bertani; MeOH, methanol.

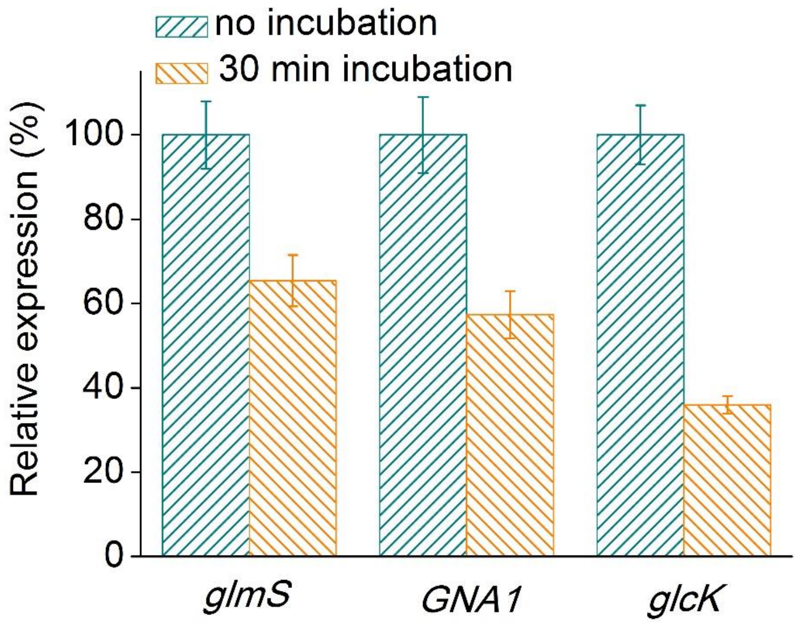

Supplementary Figure 5. Effects of expression of glucokinase and GlcNAc pathway enzymes. Quantitative real-time PCR was used for analyses of expression of glucokinase and GlcNAc pathway enzymes at transcriptional level during 30 min incubation without carbon source. Triplicate experiments were done for relative gene expression assay, error bars represent standard deviation.

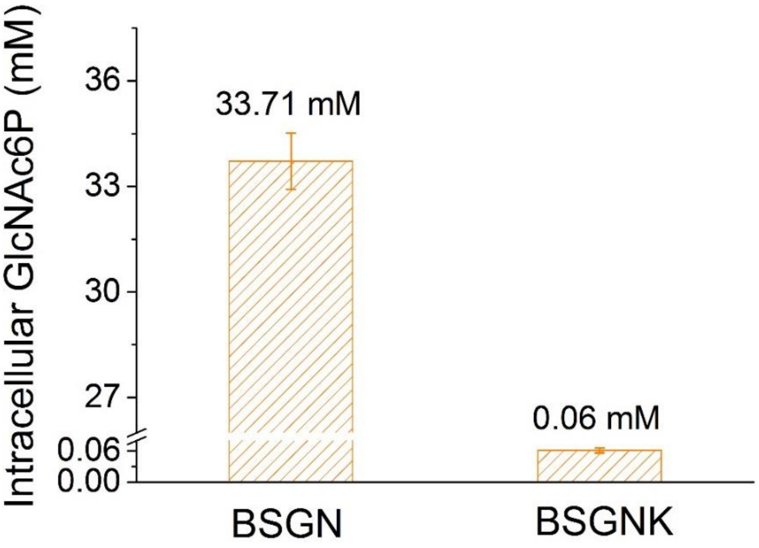

Supplementary Figure 6 Comparison of steady state intracellular GlcNAc6P concentration of BSGN and BSGNK (BSGN with *glcK* deletion). Error bars denote standard deviations of metabolite concentrations of triplicate experiments.

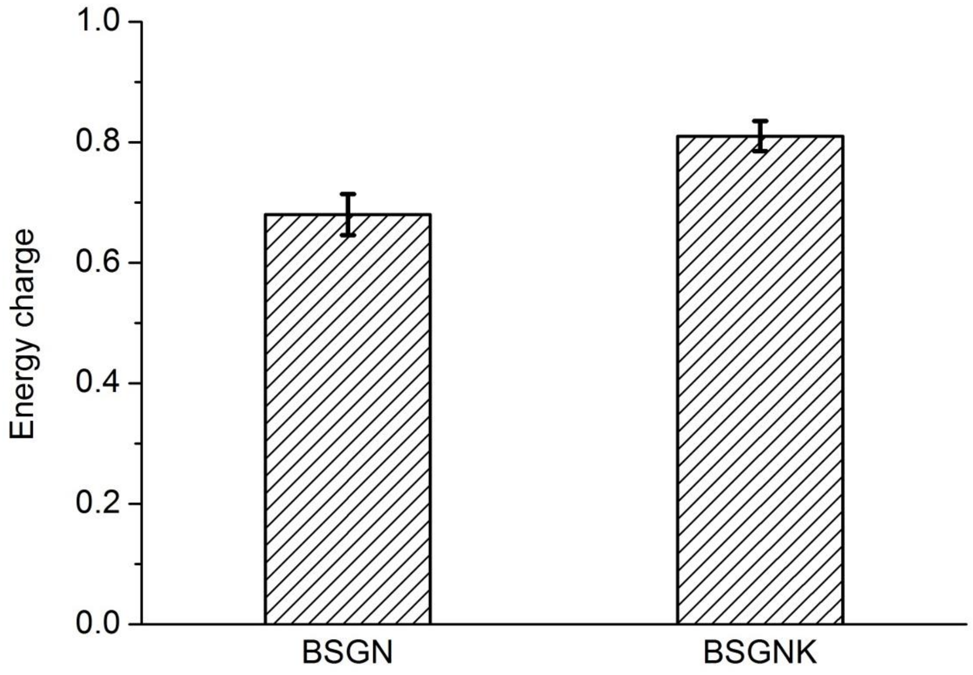

Supplementary Figure 7 Comparison of steady state energy charge of BSGN and BSGNK (BSGN with *glcK* deletion). Error bars denote standard deviations of triplicate experiments.

Supplementary Table 1 GlcNAc production and cell growth of BSGN and BSGNK

Strain and medium	GlcNAc titer (mg/L)	GlcNAc yield on glucose (mg/g glucose)	GlcNAc productivity (mg/L/h)	Specific GlcNAc production rate (mg/g DCW/h)	Specific cell growth rate (h^{-1})	Dry cell weight yield on glucose (mg/g glucose)
BSGN in complex medium with 40 g/L glucose	3550	88.8	118.3	188.0	0.40	188.8
BSGN in minimal medium with 2 g/L glucose	135	65.0	4.0	32.6	0.07	61.3
BSGNK in minimal medium with 2 g/L glucose	295	147.5	9.2	33.2	0.15	138.3

Supplementary Table 2 Physiology of *B. subtilis* 168, BSGN, BSGN-Pfk*, and BSGN-GS

Strain names	Specific cell growth rate (h^{-1})	Specific glucose uptake rate (mmol/g DCW/h)	Specific GlcANc production rate (mmol/g DCW/h)
B. subtilis 168	0.460±0.025[a]	6.411±0.251	-[b]
BSGN	0.078±0.012	1.898±0.112	0.248±0.022
BSGN-Pfk*	0.071±0.014	1.666±0.145	0.254±0.031
BSGN-GS	0.067±0.015	2.747±0.201	0.243±0.015

[a]Mean values and deviations obtained from three independent shake flask cultures

[b]Not detected

Supplementary Table 3 Steady state comparative metabolomic analysis of BSGN, BSGN-Pfk*, and BSGN-GS

metabolite	abbreviation	BSGN vs *B. subtilis* 168		BSGN-Pfk* vs *B. subtilis* 168		BSGN-GS vs *B. subtilis* 168	
		log2(fold change)	*p*-value	log2(fold change)	*p*-value	log2(fold change)	*p*-value
glycine	Gly	-0.883	0.008	-0.986	0.005	-0.370	0.022
alanine	Ala	-1.074	0.001	-1.029	0.003	-0.441	0.028
serine	Ser	-0.964	0.001	-1.113	0.001	-0.583	0.001
proline	Pro	-2.359	0.001	-2.550	0.002	-0.552	0.013
valine	Val	-2.012	0.001	-2.102	0.001	-0.612	0.001
threonine	Thr	-0.187	0.262	0.032	0.804	-0.613	0.001
asparagine	Asn	-1.585	0.001	-1.607	0.001	-0.598	0.001
asparate	Asp	-0.472	0.011	-0.617	0.006	-0.645	0.004
glutamine	Gln	-2.058	0.001	-2.312	0.001	-0.456	0.004
leucine	Leu	-1.671	0.001	-1.663	0.001	-0.550	0.005
lysine	Lys	-0.963	0.137	-0.908	0.148	-0.648	0.211
glutamate	Glu	-1.518	0.001	-1.763	0.001	-0.580	0.001
methionine	Met	-1.245	0.001	-1.655	0.001	-0.487	0.010
histidine	His	-0.280	0.013	-0.434	0.007	-0.161	0.066
phenylalanine	Phe	-0.467	0.031	-0.100	0.567	-0.225	0.070
arginine	Arg	-1.839	0.008	-1.686	0.004	-0.421	0.080
tyrosine	Tyr	-0.239	0.016	-0.257	0.013	-0.516	0.002
lactate	lactate	-0.368	0.229	-0.163	0.414	1.249	0.081
fumarate	fumarate	-1.440	0.001	-1.297	0.002	0.106	0.431
succinate	succinate	-1.560	0.003	-1.524	0.004	0.346	0.026
malate	malate	-0.843	0.001	-0.521	0.001	0.869	0.001
alpha ketoglutarate	alpha ketoglutarate	-1.958	0.001	-1.748	0.001	-0.195	0.080
phosphoenolpyruvate	PEP	-0.879	0.118	-0.991	0.102	-0.350	0.373
Dihydroxyacetone phosphate	DHAP	-0.041	0.796	0.048	0.784	-0.216	0.295

glycerol-phosphate	glycerol-P	-0.033	0.782	-0.072	0.516	-0.013	0.904
aconitate	aconitate	-1.735	0.001	-1.385	0.001	-0.324	0.009
shikimate	shikimate	0.403	0.405	0.312	0.289	-0.400	0.575
3-phosphoglycerate and 2-phosphoglycerate	xPG	-0.921	0.003	-1.214	0.002	-0.405	0.008
citrate-isocitrate	citrate-isocitrate	-2.188	0.006	-2.184	0.006	-0.046	0.828
isocitrate	isocitrate	-1.340	0.037	-0.534	0.183	-0.016	0.971
erythrose 4-phosphate	E4P	0.587	0.102	0.578	0.213	0.250	0.182
methylcitrate-methylisocitrate	methylcitrate-methylisocitrate	-1.571	0.004	-0.526	0.091	-1.870	0.001
panthothenate	pantothenate	-0.936	0.010	-0.919	0.013	-0.931	0.003
ribose-1-phosphate	R1P	0.065	0.808	-0.152	0.178	0.366	0.014
glucosamine-6-phosphate	GlcN6P	-3.202	0.013	-3.957	0.012	-0.043	0.831
glucose-6-phosphate	G6P	-0.754	0.009	-0.790	0.007	-0.283	0.043
fructose-6-phosphate	F6P	-1.454	0.107	-1.655	0.094	-0.977	0.164
glucose-1-phosphate	G1P	-0.874	0.008	-1.107	0.003	-0.137	0.340
fructose-1-phosphate	F1P	-0.842	0.009	-1.080	0.004	-0.154	0.289
6-phosphogluconate	6PG	-1.283	0.026	-1.595	0.016	-0.329	0.209
Sedoheptulose-7-phosphate	S7P	-0.072	0.345	-0.338	0.011	-1.495	0.196
fructose-1,6-phosphate	FBP	-0.643	0.001	-0.784	0.001	-0.401	0.005
adenosine monophosphate	AMP	-1.169	0.001	-1.474	0.001	-0.379	0.005
guanosine monophosphate	GMP	2.497	0.037	2.531	0.005	1.064	0.013
adenosine diphosphate	ADP	-1.193	0.002	-1.665	0.001	-0.616	0.007
guanosine diphosphate	GDP	-1.870	0.013	-2.248	0.012	-1.740	0.009
guanosine triphosphate	GTP	-2.051	0.012	-2.659	0.013	-1.441	0.011
adenosine diphosphate-pentose	ADP-pentose	0.149	0.615	0.229	0.482	0.551	0.234
N-acetylglucosamine-6-phosophate	GlcNAc6P	9.154	0.001	9.115	0.005	8.449	0.005

Supplementary Table 4 Significant metabolite pool size changes

**Highest fold changes**		**Lowest fold changes**	
**Metabolite**	**Fold change**	**Metabolite**	**Fold change**
GlcNAc6P	569.808±23.373	GlcN6P	0.108±0.023
GMP	5.648±1.351	Pro	0.189±0.039
NAD	1.64346±0.112	NADPH	0.201±0.005
E4P	1.503±0.248	Citrate-isocitrate	0.219±0.021
		Val	0.238±0.011
		GTP	0.241±0.036
		Gln	0.262±0.006
		Arg	0.288±0.010

Arg, arginine; E4P, erythrose 4-phosphate; GlcN6P, glucosamine-6-phosphate; Gln, glutamine; GMP, guanosine 5′-monophosphate; GTP, guanosine 5′-triphosphate; NAD, nicotinamide adenine dinucleotide; NADPH, reduced NAD^+; Pro, proline; Val, valine.

Supplementary Table 5 Metabolite concentration ranges and kinetic parameters in the GlcNAc synthesis pathway of GlcNAc-producing strain BSGN

**Metabolite**	**Range of concentration (mM)**	**Kinetic parameter**
F6P	1.28 to 2.15	K_m for GlmS 0.6 mM[26]
GlcN6P	0.03 to 0.08	K_m for Gna1 0.1 mM[28]
GlcNAc6P	12.80 to 19.80	—
Gln	1.47 to 2.33	K_m for GlmS 0.2 mM[2]
Glu	18.00 to 24.36	—

BSGN, *N*-acetylglucosamine-overproducing *B. subtilis* BSGN6-P_{xylA}-*glmS*-P_{43}-*GNA1*; F6P, fructose-6-phosphate; GlcN6P, glucosamine-6-phosphate; GlcNAc6P, *N*-acetylglucosamine-6-phosphate; Gln, glutamine; Glu, glutamate.

Supplementary Table 6 Strains and plasmids used in this study

Name	Relevant characteristics	Reference
**Strains**		
Bacillus subtilis 168	*trpC*, wild type	Lab stock
BSGN0	*ΔnagPΔgamPΔgamAΔnagAΔnagBΔldhΔpta::lox72*, overexpression of *glmS* under the control of promoter P_{xylA}	
BSGN		21
BSGN-Pfk*	BSGN derivate, site-directed mutation in native *pfk* Arg252Ala.	This study
BSGN-GS	BSGN derivate, native glutamine synthase (GS) overexpression under the control of promoter P_{43}	This study
Bacillus subtilis 168 *ΔglcK*	*Bacillus subtilis* 168 derivate, *ΔglcK::spc*	Lab stock
BSGNK	BSGN derivate, *ΔnagPΔgamPΔgamAΔnagAΔnagBΔldhΔpta::lox72 ΔglcK::spc*, overexpression of *glmS* under the control of promoter P_{xylA} and *GNA1* under the control of promoter P_{43}.	This study
**Plasmids**		
pDGREF	Amp^r, Spc^r, *mazF* cassette under the control of promoter P_{xylA}	31
pP43-GNA1	pP43NMK derivate with Gna1 clone	22
pP43-GNA1-GS	pP43-GNA1 derivate with GS clone	This study

BSGN, GlcNAc-overproducing *B. subtilis* BSGN6-P_{xylA}-*glmS*-P_{43}-*GNA1*; BSGN0, BSGN6-P_{xylA}-GlmS; GlcNAc, *N*-acetylglucosamine; Gna1, GlcN6P *N*-acetyltransferase; GS, glutamine synthase; Pfk, 6-phosphofructokinase.

Supplementary Table 7 Primers used in this study

Name	Sequence
AL-F	5'-ATGAAACGAATAGGGGTATTAACGAGC-3'
AL-R	5'-TTTTAAGTGTTACCCCTATAAGTTAGGGAGAACCTC*CGG*CCTGGATATGGC-3'
AZ-F	5'-GCCATATCCAG*GCC*GGAGGTTCTCCCTAACTTATAGGGGTAACACTTAAAAAAGAA-3'
AZ-R	5'-GGAGAACCTCC*GGC*CTGGATATGGCTTATAATTTTTTTAATCTGTTATTTAAATAGTTTATAG-3'
AR-F	5'-AAATAACAGATTAAAAAAATTATAAGCCATATCCAG*GCC*GGAGGTTCTCC-3'
AR-R	5'-TTGAGTGTTCCGTTGTTTAATACTTTTGT-3'
GNA1-F	5'-ATTATGTAAAATATAAAGTGATAGCGGTACCATTATAGGTAAGAGAGGAATG-3'
GNA1-R	5'-CTTTGCCATAGATCCTTCCTCCTTTTTATTTCCGAATCTGCATTTCC-3'
GS-F	5'-AAATGCAGATTCGGAAATAAAAAGGAGGAAGGATCTATGGCAAAGTACACTAGAGAAGATATC-3'
GS-R	5'-AAACAGCTATGACCATGATTACGCCTTAATACTGAGACATATACTGTTCGC-3'
V-F	5'-GGAAATGCAGATTCGGAAATAAGGCGTAATCATGGTCATAGCTG-3'
V-R	5'-TTCCTCTCTTACCTATAATGGTACCGGCGTAATCATGGTCATAGCTG-3'
Glck-F	5'-CAGAGCGGCAGCTGTCCT-3'
GlcK-R	5'-CTGCTTCCGAGATCACCCATA-3'
Primers for quantitative real-time PCR	
16s-RT-F	5'-GCGTGCTCCGTTCTATTC-3'
16s-RT-R	5'-ACTCTTTCACAAGCCATTCC-3'
glmS-RT-F	5'-GCTGTAGCGGAAGCGGACC-3'
glmS-RT-R	5'-CAGAAGCGGCATGTTGTAGGAG-3'

GNA1-RT-F	5’-AGTAAACTCCAAGTATCAG-3’
GNA1-RT-R	5’-TTCTCATCGCAATCTAAA-3’
glck-RT-F	5'-ACGATTACTGTCACAATT-3'
glck-RT-R	5'-TTTCATAAACCACTCCTG-3'

Italicized and underlined letters indicate mutation sites and homologous sequences for fusion polymerase chain reaction, respectively.
